# Physicians’ approach to end of life care: comparison of two tertiary care university hospitals in Lebanon

**DOI:** 10.1186/s12909-021-03022-x

**Published:** 2021-11-25

**Authors:** George Dabar, Imad Bou Akl, Mirella Sader

**Affiliations:** 1 Pulmonary and Critical Care Division, Hotel Dieu de France Hospital, Saint Joseph University, Boulevard Alfred Naccache Achrafieh, PO Box 166830, Beirut, Lebanon; 2 Pulmonary and Critical Care Division, American University of Beirut Medical Center, Beirut, Lebanon; 3 Anesthesia and Critical Care Division, Hotel Dieu de France Hospital, Saint Joseph University, Beirut, Lebanon

**Keywords:** End-of-life decisions, Terminal care, Physicians, Attitude, Medical culture

## Abstract

**Background:**

The care of terminally ill patients is fraught with ethical and medical dilemmas carried by healthcare professionals. The present study aims to explore the approaches of Lebanese attending physicians towards palliative care, end of life (EOL) care, and patient management in two tertiary care university hospitals with distinct medical culture.

**Methods:**

Four hundred attending physicians from the American University of Beirut Medical Center (AUBMC) and Hotel Dieu de France (HDF) were recruited. Participants were Medical Doctors in direct contact with adult patients that could be subject to EOL situations providing relevant demographic, educational, religious as well as personal, medical or patient-centric data.

**Results:**

The majority of physicians in both establishments were previously exposed to life-limiting decisions but remains uncomfortable with the decision to stop or limit resuscitation. However, physicians with an American training (AUBMC) were significantly more likely to exhibit readiness to initiate and discuss DNR with patients (*p*<0.0001). While the paternalistic medicinal approach was prevalent in both groups, physicians with a European training (HDF) more often excluded patient involvement based on family preference (*p*<0.0001) or to spare them from a traumatic situation (*p*=0.003). The majority of respondents reported that previous directives from the patient were fundamental to life-limiting decisions. However, the influence of patient and medical factors (e.g. culture, religion, life expectancy, age, socioeconomic status) was evidenced in the HDF group.

**Conclusion:**

Early physician-initiated EOL discussions remain challenged in Lebanon. Paternalistic attitudes limit shared decision making and are most evident in European-trained physicians. Establishing a sound and effective framework providing legal, ethical and religious guidance is thus needed in Lebanon.

## Introduction

The rising incidence of incurable populations spurred research into the conceptualization and characterization of optimal end-of-life (EOL) planning and enactment. Delivering optimal ethical care has become a prevailing concern in healthcare systems strained in the life-limiting context of older age and incurable chronic illness [1]. In this regard, palliative and terminal care rises in prominence as an avenue for cost-effective improved EOL medical care and quality of living [2]. EOL decisions involve consideration of preferences and priorities in terms of life prolonging or life limiting interventions, palliative treatment as well as preferred care/death settings.

Theoretically, terminal care implicates the incorporation of a premeditated medical, spiritual and psychological supportive niche for patients and their families facing life-limiting conditions. It thus falls to primary care physicians to assess patients’ situations and establish optimal care strategies to limit the physical, emotional, psychological and financial burden of EOL situations on both patients and their caregivers. However, the care of terminally ill patients is fraught with ethical and medical challenges in actual practice. In the absence of advance directives from patients, healthcare professionals are often left with the daunting task of initiating and authorizing life-determining decisions. Physicians thus become surrogates for EOL decisions, funneling them through considerations of what the patient would have chosen if mentally competent (substituted judgment) or optimal patient-centric outcomes (best interest of the patient) [3].

From a caregiver perspective, a procession of deliberation, resolution and decision enactment underlies EOL decisions [4]. In the Netherlands, patient relatives implicated in EOL decisions actually seemed to exhibit more permissive attitudes towards death than physicians [5]. However, healthcare professionals, such as physicians, remain among the primary influencers of the thoughts and attitudes of patient relatives and caretakers facing decisions to withhold or stop life-sustaining medical interventions [4]. Nurses and physicians can optimize EOL experiences for both patients and their caretakers [6] and resolve decisional and religious conflicts that could arise among families [7].

Regardless, the opportunity to engage in EOL decisions before terminally ill patients lose their decisional capacity is often missed [8]. Physicians are generally held accountable for EOL decisions and continue to be challenged by various factors [9]. Among these, role ambiguity, communication issues and discussion initiation remain the most notable barriers to EOL discussions [9]. In fact, healthcare professionals in developing countries often lack confidence in their EOL care skills and shy away from initiating EOL discussions [10]. The need to improve care staff self-efficacy and confidence in aspects related to EOL patient care is thus evident, even in the context of developed countries [11, 12].

The subjectivity of surrogate EOL and treatment discontinuation decisions fuels considerable legal and moral dilemmas borne primarily by physicians. Physician-related factors , such as religiosity and education, thus contribute to existing patient-centric considerations and influence physician attitude towards the initiation and enactment of EOL decisions [5, 13]. In the middle east, cultural, religious, educational and clinical factors have all been reported to affect physician attitudes towards EOL considerations [14, 15]. However, evidence from the region remains limited, with much remaining to be explored and determined in terms of EOL decision making processes and their influencing factors in individual countries.

The present study thus aims to explore the approaches of Lebanese attending physicians towards palliative care, EOL care and patient management in two tertiary care university hospitals with distinct medical culture. Attitudes towards the limitation or discontinuation of active therapeutics in patients reaching EOL will be explored, in addition to the implication of the patient in the decisional process and the factors that could affect physician attitude.

## Methods

A collaborative study was conducted between two research sites: the American University of Beirut Medical Center (AUBMC) and Hotel Dieu de France (HDF). Attending physicians from the two tertiary care academic hospitals were recruited to participate in a survey of attitudes towards EOL decisions and their contributing factors. Participants were Medical Doctors of either gender (male or female) that are practicing at AUBMC or HDF. Attending physicians must have direct contact with adult patients that could be subject to EOL situations, which excluded radiologists, laboratory medicine specialists, and pediatricians from the study.

Participants provided demographic data including age, gender as well as familial educational attainment and information pertaining to religiosity. Physicians’ educational background and training setting was also explored, in addition to their attitude towards EOL care and the personal, medical or patient-centric factors that affect the ethical decision-making process in these two institutions. These include patient-related and clinical data such as the availability of advance directives, family opinion, economic considerations and patient prognosis.

### Ethical considerations and data handling

The main survey tool consisted of a questionnaire targeting around 400 attending physicians at the AUBMC, 100/200 replied and at HDF 67 out of 200 answered to our solicitation over a period of four months. Primary data was collected specifically for the research paper, and no secondary data was generated or used. Data collection was completed through a three-page hard copy or digital questionnaire distributed by the primary investigators through departmental offices or by email. Study participation was strictly voluntary. Once completed, all questionnaires were submitted to the primary investigators and anonymous data containing no identifiers was used for secondary analysis. Institutional review board approval was obtained from the HDF and AUBMC ethics committees prior to the initiation of the study and all the methods were performed in accordance with the relevant guidelines and regulations. An informed consent was obtained from all the subjects who participated in the study.

### Statistical analysis

Data were analyzed using SPSS software (IBM Corp., SPSS Statistics for Windows Version 26·0, Armonk, NY). Categorical variables were presented as frequencies and percentages and compared using the Pearson Chi square test. Continuous variables not departing from normality assumptions were presented as mean ± standard deviation (SD) and compared using the independent samples t-test. Ordinal variables and continuous variables departing from normality assumptions were expressed as median with interquartile range (1st quartile – 3rd quartile) and compared using the Mann-Whitney U test. All the statistical tests were 2-sided and the level of significance was set to 0.05.

## Results

One hundred practicing physicians from the AUBMC and 67 from HDF hospital participated in this study. Sociodemographic data revealed that study samples were comparable in some regards while showing distinct variability, as shown in Table 1. The male gender overshadowed female gender as approximately three quarters of physicians were males in both samples. Comparable age was reported in both study settings and participants were middle aged physicians aged close to 52 years on average. On a familial level, sibling distribution and the highest educational level of participants’ mothers were similar, while highest paternal educational attainment varied significantly between the two samples, with a higher proportion of post-graduate fathers reported among physicians from the AUBMC. While the degree of religiosity was comparable in both settings, the vast majority of physicians were Christian in the HDF hospital sample (98.5%), while the AUB exhibited a religiously diverse population that remained predominately formed of Christian (35.3%) and Muslim (45.9%) physicians. As for training background, the majority of physicians practicing in HDF hospital specialized in a Lebanese/French curriculum, while their counterparts in the AUBMC predominately studied between Lebanon and the United States.

**Table 1:** Sociodemographic, religious, and medical training characteristics of attending physicians from the American University of Beirut Medical Center (AUBMC) and Hotel Dieu de France (HDF) participating in the study

	AUBMC	HDF	*p*-value
Gender			
Male Female	75(75%) 25(25%)	51(76.1%) 16(23.9%)	0.869†
Age	52.8 ± 10.9	53.7 ± 8.8	0.554ɸ
Number of siblings			
0	4(4.1%)	2(3.2%)	
1	12(12.2%)	17(27%)	
2	32(32.7%)	18(28.6%)	
3	30(30.6%)	15(23.8%)	0.117‡
4 5 6 7	11(11.2%) 6(6.1%) 2(2%) 1(1%)	7(11.1%) 4(6.3%) 0(0%) 0(0%)	
Highest paternal educational attainment			
Primary Education	11(11.1%)	2(3%)	
Complementary Education	4(4%)	11(16.4%)	
Secondary Education University Degree University Post-Graduate Degree	17(17.2%) 25(25.3%) 42(42.4%)	19(28.4%) 19(28.4%) 16(23.9%)	0.033‡
Highest maternal educational attainment			
Illiterate	8(8%)	1(1.5%)	
Primary Education	6(6%)	4(6%)	
Complementary Education Secondary Education University Degree University Post-Graduate Degree	6(6%) 31(31%) 31(31%) 18(18%)	10(14.9%) 28(41.8%) 14(20.9%) 10(14.9%)	0.315‡
Religious background			
Christian	30(35.3%)	65(98.5%)	
Muslim	39(45.9%)	1(1.5%)	
Druze Atheist other none	4(4.7%) 2(2.4%) 2(2.4%) 8(9.4%)	0(0%) 0(0%) 0(0%) 0(0%)	0.000†
Degree of religiosity			
Non-believer	14(14.1%)	1(1.5%)	
Non-practicing believer Occasionally practicing believer Regularly practicing believer	18(18.2%) 32(32.3%) 35(35.4%)	11(16.7%) 35(53%) 19(28.8%)	0.435‡
Country(ies) of specialization			
Lebanon France United States Canada United Kingdom Other	73(73.7%) 5(5.1%) 60(60.6%) 6(6.1%) 12(12.1%) 3(3%)	59(88.1%) 66(98.5%) 7(10.4%) 4(6%) 0(0%) 1(1.5%)	0.019† 0.000† 0.000† 0.994† 0.003† 0.650ㅓ
Number of years spent in country of specialization:			
Lebanon France United States Canada	4[3 ;7] 1[0;2] 4[1;8.5] 3[2;5]	4[3;4] 3[2;4] 1[1;3] 4[2.5;4]	0.060 0.701δ 0.141δ 0.467δ
United Kingdom	4.5[2.5;5]	-	
Other	6[2;7]	4[4;4]	1.000δ

*p* values calculated via Pearson Chi-Square†, t test of equality meansɸ, Mann-Whitney U‡, Independent-Samples Median Testδ, Fisher’s Exact Testㅓ or as applicable. Numbers between squared brackets represents 1^st^ and 3^rd^ quartile around the median value. Significance set at *p* value <0.05

More than 80% of participating physicians in both samples reported having been previously exposed to Do-Not-Resuscitate (DNR) decisions. Uncertainty when handling DNR decisions was not frequent in both establishments (*p*=0.313). However, notable differences could be observed in the attitudes of practicing physicians in regards to EOL decisions. Physicians attending AUBMC were significantly more likely than HDF physicians to exhibit readiness to initiate and discuss DNR with patients retaining the mental competence to be involved in such decisions (68 vs 34.3%, *p*<0.0001). HDF physicians had a higher preference towards patient-initiated discussions regarding DNR directives when compared to their counterparts in AUBMC (43.3 vs 21%, *p*=0.002). The hesitancy of HDF physicians to initiate DNR decision-making was also reflected in their apparent preference of delaying DNR discussions until serious clinical deterioration and excluding the patient from these considerations.

A notable number of physicians in both AUBMC and HDF remains uncomfortable with the decision to stop or limit resuscitation. While some physicians perceived the patients to not want to be included in the decision, lack of time and refusal to discuss the prognosis with patients infrequently limited patient inclusion in both establishments. The paternalistic medicinal approach was prevalent among respondents from both groups with more than 30% of physicians in either sample believing they are better placed than the patient to make the DNR decision. In addition to lack of physician comfort, the most frequent barriers to patient participation in DNR decision in AUBMC were reported to be acting upon family wishes to exclude patient from decision (46.2%), and personal belief that the experience will be traumatic to the patient.

While generally comparable, the barriers preventing the inclusion of patients in DNR decisions showed some distinct variability between study group. Physicians from the HDF were significantly more likely to refuse patient involvement based on family wishes than AUBMC physicians (71.9 vs 46.2%, *p*<0.0001). Moreover, HDF physicians were more likely to exclude patients from the decision in order to spare them from a traumatic and anxiety-inducing situation (42.9 vs. 32.7%, *p*=0.003), further highlighting the paternalistic predilection of this group. While approximately 15% of physicians attending AUBMC considered patient ethnicity, culture, religion or other patient characteristic when handling DNR decisions, HDF physicians were more likely to consider them limiting factors in regards to patient participation (24.6%, *p*=0.016)

Table 2 also presents perceptions of attending physicians regarding the role or implication of medical staff, be it the attending physician or the medical team in general, on DNR decisions. Physicians from either establishment agreed on the importance of the attending physician’s opinion when it comes to DNR decisions. In fact, close to 40% of physicians accorded high importance to the attending physician’s opinion, while 28.6 and 34.8% of respondents from AUBMC and HDF, respectively, thought it was fundamental. On the other hand, physicians did not exhibit comparable opinions regarding the role of the medical team in DNR decisions. Physicians from the HDF seemed to accord more value to the medical team’s opinion than their counterparts in AUBMC, where approximately 16% of physicians thought it was of little importance.

**Table 2:** Physician experience, attitude and barriers to EOL decisions, such as DNR, among practicing physicians in the American University of Beirut Medical Center (AUBMC) and Hotel Dieu de France (HDF) participating in the study

	AUBMC	HDF	*p*-value
Prior participation in DNR decision			
No Yes	15(15%) 85(85%)	8(11.9%) 59(88.1%)	0.574†
Attitude toward DNR decision			
- Ready for physician-initiated discussion with mentally competent patient.	68(68%)	23(34.3%)	0.000†
- Ready for patient-initiated discussion with mentally competent patient	21(21%)	29(43.3%)	0.002†
- Would discuss upon serious clinical deterioration without involving the patient	12(12%)	16(23.9%)	0.044†
- Uncertain about what to do	5(5%)	6(9%)	0.313†
Criteria that preclude patient participation in decision			
- Not comfortable with the decision	19(36.5%)	19(33.3%)	0.157†
- Lack of time on the part of the attending physician	4(7.7%)	7(12.3%)	0.100†
- Respect the wishes of the family who refuse to involve the patient.	24(46.2%)	41(71.9%)	0.000†
- Refusal to discuss prognosis with patient	7(13.5%)	6(10.5%)	0.644†
- Refusal to inform the patient of his diagnosis	10(19.2%)	3(5.3%)	0.192†
- Personal belief that the patient does not want to be engaged in these discussions	12(23.1%)	10(17.5%)	0.584†
- Personal conviction that the discussion is traumatic and a source of anxiety for the patient	17(32.7%)	25(43.9%)	0.003†
- Physician is better placed through the doctor-patient relationship to determine the course of treatment in the patient's best interest	18(34.6%)	18(31.6%)	0.172†
- Certain personal characteristics of the patient (ethnicity, culture, religion, age, socioeconomic level	8(15.4%)	14(24.6%)	0.016†
Opinion of physician in charge			
Not important at all Somewhat important Important Very important Fundamental	2(2%) 7(7.1%) 22(22.4%) 39(39.8%) 28(28.6%)	1(1.5%) 3(4.5%) 10(15.2%) 29(43.9%) 23(34.8%)	0.177‡
Opinion of medical team (Physicians)			
Not important at all Somewhat important Important Very important Fundamental	0(0%) 16(16.2%) 28(28.3%) 35(35.4%) 20(20.2%)	1(1.5%) 2(3%) 15(22.4%) 30(44.8%) 19(28.4%)	0.018‡

In regards to patient-related factors (Table 3), the opinion of a patient’s family was considered very important by 44 and 31.3% of physicians from the AUBMC and HDF, respectively. More than 70% of both samples agreed that previous directives from the patient were fundamental to the DNR or active treatment discontinuation decisions, with no physicians considering it inconsequential. However, significant inconsistencies could be observed in medical and clinical aspects pertaining to these EOL decisions. In fact, physicians from the HDF were more concerned about patients’ life expectancy when formulating their attitude towards EOL decisions. This was evidenced on the level of both pre- and post-admission life expectancy, while the same variations were not extended to patients’ quality of life. Concerning the latter, comparable results were reported in both samples, where high importance was accorded to pre- and post-admission quality of life.

**Table 3:** Patient- and clinical-related factors affecting DNR decisions, as reported by attending physicians from the American University of Beirut Medical Center (AUBMC) and Hotel Dieu de France (HDF) participating in the study

	AUBMC	HDF	*p*-value
Opinion of the family			
Not important at all Somewhat important Important Very important Fundamental	1(1%) 6(6%) 21(21%) 44(44%) 28(28%)	1(1.5%) 8(11.9%) 14(20.9%) 21(31.3%) 23(34.3%)	0.878‡
Previous request by patient			
Not important at all Somewhat important Important Very important Fundamental	0(0%) 0(0%) 6(6%) 16(16%) 78(78%)	0(0%) 1(1.5%) 3(4.5%) 16(23.9%) 47(70.1%)	0.281‡
Life expectancy before admission			
Not important at all Somewhat important Important Very important Fundamental	4(4%) 21(21.2%) 33(33.3%) 22(22.2%) 19(19.2%)	0(0%) 3(4.5%) 23(34.8%) 25(37.9%) 15(22.7%)	0.006‡
Life expectancy after admission			
Not important at all Somewhat important Important Very important Fundamental	1(1%) 13(13.1%) 27(27.3%) 34(34.3%) 24(24.2%)	0(0%) 3(4.5%) 8(12.1%) 27(40.9%) 28(42.4%)	0.001‡
Quality of life before admission			
Not important at all Somewhat important Important Very important Fundamental	1(1%) 13(13%) 26(26%) 38(38%) 22(22%)	0(0%) 6(9.1%) 15(22.7%) 31(47%) 14(21.2%)	0.434‡
Quality of life post admission			
Not important at all Somewhat important Important Very important Fundamental	2(2%) 5(5%) 16(16%) 45(45%) 32(32%)	1(1.5%) 4(6.1%) 13(19.7%) 26(39.4%) 22(33.3%)	0.847‡
Age of the patient			
Not important at all Somewhat important Important Very important Fundamental	17(17.2%) 36(36.4%) 19(19.2%) 16(16.2%) 11(11.1%)	3(4.5%) 10(15.2%) 21(31.8%) 26(39.4%) 6(9.1%)	0.000‡
Socioeconomic status			
Not important at all Somewhat important Important Very important Fundamental	72(72%) 19(19%) 7(7%) 2(2%) 0(0%)	32(48.5%) 16(24.2%) 8(12.1%) 9(13.6%) 1(1.5%)	0.001‡
Gravity score			
Not important at all Somewhat important Important Very important Fundamental	3(3.1%) 28(29.2%) 39(40.6%) 20(20.8%) 6(6.3%)	0(0%) 14(21.2%) 26(39.4%) 18(27.3%) 8(12.1%)	0.042‡
Economic constraints			
Not important at all Somewhat important Important Very important Fundamental	35(35%) 36(36%) 20(20%) 7(7%) 2(2%)	13(19.7%) 26(39.4%) 18(27.3%) 7(10.6%) 2(3%)	0.031‡
Gender of the patient			
Not important at all Somewhat important Important Very important Fundamental	100(100%) 0(0%) 0(0%) 0(0%) 0(0%)	66(100%) 0(0%) 0(0%) 0(0%) 0(0%)	1.000‡

While gender of the patient had no implication in EOL decisions across study groups, significant differences in the perception of age and other factors could be discerned. In the AUBMC, physicians were less concerned about patient age and gravity score when formulating their EOL decisions when compared to their counterparts in HDF. Actually, almost 40% of attending physicians in HDF reported patient age to be very important in EOL decisions, compared to 16.2% in the AUBMC sample. AUBMC physicians were similarly less concerned about socioeconomic status and economic constraints. While the majority of physicians attending AUBMC thought socioeconomic status and economic issues were of little to no importance, 27.3% of HDF physicians reported economic constraints to be important influencers of their decisions.

## Discussion

To the best of our knowledge, the present study was the first to explore the approach of Lebanese physicians towards EOL decisions. in general, Lebanese physicians seemed to be well-versed in EOL situations, such as Do-Not-Resuscitate (DNR) decisions. Uncertainty when handling DNR decisions was infrequent in both studied establishments. Regardless, a clear preference towards patient-initiated and delayed EOL discussions could be observed in the French establishment (European training). Literature shows that hesitancy in EOL care might not be restricted to particular countries or cultures. Despite the increasing legalization of EOL practices such as euthanasia and physician assisted suicide, physician and public support of these interventions remains tentative [16]. In fact, such interventions remain widely debated and remain rarely practiced even in countries and American states where they have been legalized [17].

Consistently with our study, reluctance to initiate EOL discussions seems rampant among European physicians [18]. In fact, physicians were reported to be often ready to respond to patient requests and initiatives but hesitate to take the initiate upon themselves [18]. A study from France has shown that the reluctance of physicians towards EOL practices, such as physician assisted death and treatment withdrawal, could be mediated by their lack of training and perceived inadequate professional competence in this regard [19]. It thus becomes difficult for physicians to discern the optimal ethical and legal choice, which promotes feelings of uncertainty and ambivalence towards EOL care practices [20].

Providing training is thus critical to facilitate the navigation of the moral, ethical and practical dilemmas posed by EOL care. Evidence from the Middle East revealed that training background accounted for the variability of physician’s interpretation of concepts related to EOL care, such as DNR orders [14], while inadequate training prevented effective EOL discussions [21]. Education targeting terminal care discussions could not only boost the confidence of medical professionals implicated in the care of terminally ill patients but also emphasize the importance of early EOL discussions [22]. Updating available course offerings could also help to promote patient-centric considerations of preferences in terms of EOL care and dying. Training should address the need for early individualized approaches to EOL discussion, and their integration into existing care structures [23], which would ultimately promote favorable attitudes towards EOL practices among physicians [18].

Regardless of the establishment they practiced in, Lebanese physicians reported being uncomfortable with the decision to stop or limit resuscitation. EOL care is an immature concept in Lebanon with little to no guidance provided to physicians in this regard. This could explain the attitude of Lebanese physicians seeing as insecurities among medical care staff often lead to delayed discussions with either patients or their relatives [23]. Shared decisions in the context of EOL also reflect societal, educational, cultural, and religious values in both patients and physicians. In our study, religiosity was comparable in both study samples. Religiosity was previously shown to carry a significant negative influence on the attitude of physicians towards EOL and physician-assisted dying practices [18]. This was emphasized in the Middle East, where religion was an important consideration in physicians’ DNR decisions despite their exposure to Western curriculums [14]. When provided, EOL care guidance in this region must account for religious beliefs considering that its population remains deeply entrenched in religion. EOL practices such as euthanasia and assisted suicide are actually forbidden in Islam. Regardless, Islamic guidance in the form of Fatwa has accorded medical professionals with the ability to undertake DNR decisions in hopeless medical cases irrespective of family and patient preferences [24]. This could account for studies reporting that Muslim religiosity does not affect views of the religious feasibility of DNR decisions [25] and palliative medicine among physicians [26], which highlights the need to account for the capacity of other factors, such as country of origin and country of practice, to overshadow religious beliefs [25].

Paternalistic intervention overrides individual autonomy in our study despite physicians from both samples having been exposed to western curriculums and spent a considerable portion of their specialization abroad. Similarly, almost half of physicians surveyed in the middle east preferred having the ultimate authority in DNR decisions [14]. Even in Western countries, it remains debated whether palliative care and EOL practices alleviate suffering or cause it. Physicians thus continue to exhibit paternalistic and death-denying predilections, preferring life-prolonging and curative approaches to patient care [27]. Regardless, Anglo-Saxon medical professionals lean towards patient autonomy, while French physicians considering EOL decisions remain constrained by rationalistic, paternalistic, and religious traditions in addition to fears of legal prosecution [28].

As opposed to patient autonomy-oriented states, considerations of patient autonomy are disregarded in most countries in favor of delivering culturally sensitive medical care [29]. Consistently, physicians exposed to the French curriculum were significantly more likely to exclude patients from EOL decisions due to familial preferences and cultural (e.g. religion, ethnicity) considerations. This translates into physicians believing they are better placed to make the DNR decision than the patient, or even families and medical staff as was reported in Hungary [30]. Cultural and religious backgrounds most likely contribute to this paternalistic approach to EOL, seeing as similar issues are observed in religious societies such as India [31]. In Saudi Arabia, cultural factors and lack of understanding from the patient and family side were reported as notable barriers to the initiation of DNR orders and effective EOL discussions, respectively [21].

Regardless, it should be noted that paternalistic EOL practices were reported in physicians both study groups. Personal physician characteristics have been previously shown to affect attitudes towards EOL decisions [32]. In our study, physicians were predominately male and middle aged. This could have further potentiated paternalistic behavior seeing as older Arab physicians were actually reported to be more likely to perceive DNR orders as taboo and were less likely to undertake them [15]. Another of the frequent barriers to patient participation in DNR decision in our study was the personal belief that the experience will be traumatic to the patient. Avoidance of the negative psychological sequelae of EOL discussion among patients seem to be a frequent deterrent among medical care staff [22]. Physicians were reported to recognize the psycho-existential suffering that terminal illnesses carry and integrate them into their EOL decisions, albeit in variable manners [18].

Regardless, physicians from the French university were more likely to exclude patients from the decision in order to spare them from a traumatic and anxiety-inducing situation (42.9 vs. 32.7%, *p*=0.003), further highlighting the paternalistic predilection of this group.

Graduates from the French university were more significantly concerned by patient and clinical factors, such as prognosis, age, and gravity of disease in EOL situations, reflecting their preference of the nonmaleficence ethical approach. They also seemed more concerned about patient age, socioeconomic status and economic constraints when formulating their EOL decisions when compared to their counterparts in the American curriculum. Poor prognosis actually remains a prevalent facilitator of DNR orders and treatment discontinuation decisions among physicians in different contexts [33, 34]. Consistently, European physicians seemed to consider EOL practices such as euthanasia and forgoing artificial nutrition and hydration more favorably should patients be known to be near death [18]. This reflects the preference of Saudi Arabian outpatients, who preferred to discuss DNR directives when suffering from illness [35]. A study from Saudi Arabia showed that while medical expenses were not prevailing concerns in EOL decisions among physicians, the dignity and prognosis of elderly patients played an important role in issuing DNR orders [36]. In another study, Saudi Arabian physicians reported the implication of debilitating patient diagnosis, particularly neurological diseases, in determining the likelihood of DNR orders being issued [15]. When facing EOL situations, it seems that preparing for death while managing pain and disease symptoms are prioritized by both physicians and patients, with the latter exhibiting additional concerns of not being a burden on the family and coming to peace with God [37].

## Conclusions

While EOL decisions are prevalent in Lebanese hospitals, they are differentially approached by attending physicians. Early physician-initiated EOL discussions are challenged in both American and French institutions in Lebanon by several clinical, personal and patient-related considerations. Paternalistic attitudes frequently preclude shared decision making and are most evident in French-oriented physicians. Establishing a sound and effective framework providing legal, ethical and religious guidance is thus needed in Lebanon. Supported by educational interventions promoting physician confidence and emphasizing the integration of patient autonomy, such strategies should ensure the improvement and facilitation of EOL care.

## Data Availability

The datasets used and/or analysed during the current study are available from the corresponding author on reasonable request.

## Abbreviations

AUBMC: American University of Beirut Medical Center
DNR: Do-Not-Resuscitate
EOL: End of Life
HDF: Hôtel Dieu de France
